# An anoikis-related four-gene prognostic signature and functional characterization of ANXA5 in laryngeal squamous cell carcinoma

**DOI:** 10.3389/fimmu.2026.1941275

**Published:** 2026-09-15

**Authors:** Diandian Ba, Wenwei Wang, Yitao Tang

**Affiliations:** 1 Department of Hematology, The Affiliated Cancer Hospital of Zhengzhou University, Henan Cancer Hospital, Zhengzhou, Henan, China; 2 Department of Pathology and Lab Medicine, Shandong Cancer Hospital and Institute, Shandong First Medical University and Shandong Academy of Medical Sciences, Jinan, Shandong, China; 3 Department of Otolaryngology-Head and Neck Surgery, Renji Hospital, Shanghai Jiao Tong University School of Medicine, Shanghai, China

**Keywords:** anoikis, laryngeal squamous cell carcinoma, prognosis, prognosis prediction, tumor immune microenvironment

## Abstract

**Background:**

Laryngeal squamous cell carcinoma (LSCC) remains one of the most lethal malignancies of the head and neck region. Tumor metastasis, the primary cause of treatment failure, is intimately linked to the evasion of anoikis. Yet the molecular drivers of this process in LSCC are incompletely understood. This study systematically investigates the prognostic and functional landscape of anoikis-related genes (ARGs) in LSCC, with an emphasis on their interplay with the tumor immune microenvironment and therapeutic vulnerability.

**Methods:**

RNA-sequencing profiles and clinical data from the TCGA-LSCC cohort were analyzed, and candidate anoikis-related genes (ARGs) were curated from the GeneCards and Harmonizome databases. Differentially expressed and prognosis-associated ARGs were screened using limma and univariate Cox regression. A four-gene prognostic signature was constructed in the training set using LASSO-Cox and multivariate Cox regression and internally evaluated in the testing set. The resulting anoikis-related risk score was assessed in relation to survival, clinicopathological variables, immune characteristics, pathway enrichment, and predicted drug response. Single-cell RNA-sequencing data were used to characterize the cellular distribution of the signature genes. ANXA5 was prioritized for experimental evaluation because it had the largest positive model coefficient among the risk-associated genes, showed tumor-associated dysregulation, and had biological relevance to anoikis-related processes. Its effects were examined in TU686 cells using expression, cell-viability, EMT-marker, apoptosis, and wound-healing assays.

**Results:**

The ARG-based signature effectively stratified LSCC patients into high- and low-risk groups with significantly different overall survival (p < 0.001). High anoikis-related risk score correlated with an immunosuppressive TME, suggesting a permissive milieu for immune evasion. Drug sensitivity analysis revealed that high-risk patients were more responsive to certain tyrosine kinase inhibitors but resistant to conventional chemotherapeutics, highlighting subtype-specific therapeutic opportunities. Among the ARGs, *ANXA5* emerged as a hub gene with strong prognostic power. *In vitro* assays demonstrated that ANXA5 exerts oncogenic functions in LSCC. Following ANXA5 knockdown, we detected suppressed proliferative activity, differential expression of EMT signature genes, elevated apoptosis rate and weakened migratory ability in laryngeal squamous carcinoma cells. Collectively, these preliminary results suggest that ANXA5 is likely implicated in regulating proliferation, mesenchymal molecular traits, cell survival and migratory behaviors of laryngeal squamous carcinoma cells.

**Conclusion:**

The four-gene anoikis-related signature showed prognostic stratification within the TCGA-LSCC cohort and was associated with differences in the tumor immune microenvironment. ANXA5 may represent a candidate prognostic biomarker and a biologically relevant gene for further mechanistic investigation; however, external validation and additional functional studies are required.

## Introduction

Laryngeal squamous cell carcinoma (LSCC), one of the most common malignant tumors of the head and neck, severely impairs patients’ vocalization, breathing, and swallowing functions, posing a significant threat to quality of life and survival (1–3). Its pathogenesis is closely associated with long-term smoking, alcohol consumption, human papillomavirus infection, and environmental factors, with a predilection for middle-aged and elderly males (4). With advancements in diagnostic techniques—particularly the widespread use of electronic laryngoscopy and imaging modalities—the detection rate of early-stage LSCC has markedly increased, thereby creating favorable conditions for larynx-preserving surgical approaches (5). Nevertheless, the treatment of advanced cases remains highly challenging. This article aims to explore the pathogenesis of laryngeal cancer and to identify potential candidate biomarker.

Anoikis, a specialized apoptotic process triggered by loss of cell anchorage to the extracellular matrix (ECM), functions as a natural defense against ectopic cell growth and metastasis in normal tissues (6–8). Upon detachment, epithelial cells activate pro-apoptotic pathways (via caspase activation and mitochondrial dysfunction) to eliminate displaced cells and preserve tissue architecture. In contrast, cancer cells often develop anoikis resistance, enabling prolonged survival in anchorage-independent conditions, intravasation into the bloodstream or lymphatics, and eventual metastatic colonization (9). This acquired resistance is mediated by aberrant activation of survival signals (PI3K/AKT, MAPK/ERK, integrin signaling), upregulation of anti-apoptotic proteins, and epithelial-mesenchymal transition (EMT), which collectively promote detachment tolerance, invasiveness, and therapeutic evasion (10, 11). In HNSCC, including LSCC, anoikis resistance has been strongly linked to aggressive tumor behavior, enhanced metastatic potential, and unfavorable prognosis, as evidenced by transcriptomic studies demonstrating altered expression of anoikis-related genes in detached or suspension-cultured cells (12–15).

Emerging bioinformatics approaches have highlighted the prognostic and biological significance of anoikis-related genes (ARGs) across multiple malignancies, including HNSCC (15–17). Several studies have constructed ARG-based signatures that stratify patients by risk, correlate with immune microenvironment features (infiltration levels and checkpoint expression), and predict responses to immunotherapy or chemotherapy (18–21). Recent computational oncology studies have increasingly integrated bulk transcriptomics, single-cell analysis, machine-learning-based prognostic modeling, immune landscape characterization, and therapeutic response prediction to identify clinically relevant molecular signatures across malignancies (22). For example, neddylation-related and NK cell-related gene signatures have been reported to predict prognosis, immune infiltration, and potential treatment response in cutaneous melanoma (23, 24). In LSCC, lactate-related gene signatures have also been shown to provide prognostic information and experimental evidence for metabolic vulnerability (25). These findings support the feasibility of constructing biologically interpretable gene signatures that connect molecular dysregulation, tumor immune microenvironment remodeling, and therapeutic sensitivity.

The present study addresses these gaps by systematically identifying anoikis-related differentially expressed genes (ARDEGs) in LSCC, leveraging GSE290927 for anoikis enrichment scoring and TCGA-LSCC for expression profiling, survival analysis, and signature construction. We developed and validated a four-gene prognostic signature (ANXA5, LCK, PPARG, SERPINA1), evaluated its independent prognostic power alongside clinicopathological factors, explored links to immune infiltration, pathway enrichment, and drug sensitivity, and delineated gene expression patterns at single-cell level. Additionally, we performed *in vitro* knockdown experiments on ANXA5—a key risk-associated gene—to confirm its functional contributions to proliferation, EMT, apoptosis resistance (anoikis evasion), and migration in LSCC cells. These multifaceted analyses aim to enhance understanding of anoikis dysregulation in LSCC pathogenesis, offer a clinically relevant prognostic tool, and identify promising targets for precision therapy in this challenging malignancy.

## Methods

### Data acquisition and processing

RNA-sequencing (RNA-seq) data and corresponding clinicopathological information for laryngeal squamous cell carcinoma (LSCC) were downloaded from The Cancer Genome Atlas (TCGA; https://portal.gdc.cancer.gov/) on June 16, 2025. After excluding samples without survival information or available clinical follow-up, 12 adjacent non-cancerous tissue samples, 111 LSCC tumor samples, and clinical data from 111 LSCC patients were included in the subsequent analyses. Candidate anoikis-related genes (ARGs) were retrieved from the GeneCards database (https://www.genecards.org) using the search term “anoikis”, with a relevance score > 0.4 as the screening criterion. Additional ARGs were obtained from the Harmonizome database (https://maayanlab.cloud/Harmonizome/) using the same keyword. The genes obtained from the two databases were merged, and duplicate genes were removed. For candidate ARGs available in the TCGA-LSCC expression matrix, their expression levels were extracted for subsequent differential expression analysis.

### Identification of differentially expressed ARGs

Differential expression analysis between LSCC tumor tissues and adjacent non-cancerous tissues was performed using the “limma” R package. Genes with |log2 fold change| > 1.0 and adjusted P < 0.05 were considered differentially expressed genes (DEGs). Differentially expressed ARGs were then screened by intersecting DEGs with the ARG list. Univariate Cox regression analysis was conducted to identify prognosis-associated anoikis-related differentially expressed genes using overall survival (OS) data, with P < 0.05 as the significance threshold. Coexpression relationships among candidate genes were visualized by correlation network analysis.

### Construction and evaluation of the anoikis-related prognostic signature

The 111 LSCC patients were randomly divided into a training set (n = 67) and a testing set (n = 44) at an approximate ratio of 6:4. In the training set, prognosis-associated ARGs were further reduced using least absolute shrinkage and selection operator (LASSO)-Cox regression with 10-fold cross-validation to avoid overfitting. Multivariate Cox regression analysis was then applied to construct the final prognostic model. The model-derived score was consistently termed the 2E and was calculated as follows: anoikis-related risk score = 0.657956 × ANXA5 expression + 0.338447 × PPARG expression + 0.451612 × SERPINA1 expression − 0.863213 × LCK expression. This four-gene model-derived score is distinct from the single-cell anoikis module score calculated using AddModuleScore.

The final model consisted of four genes: ANXA5, PPARG, SERPINA1, and LCK. The model coefficients were 0.657956 for ANXA5, 0.338447 for PPARG, 0.451612 for SERPINA1, and -0.863213 for LCK.

Patients in the training set were classified into high- and low-risk groups according to the median anoikis-related risk score of the training set, and the same cutoff was applied without re-estimation to the testing set and the entire TCGA-LSCC cohort. Kaplan-Meier survival curves with log-rank tests were generated using the “survival” and “survminer” R packages. Receiver operating characteristic (ROC) curves and time-dependent ROC curves were generated using the “timeROC” R package to evaluate the predictive performance for 1-, 3-, and 5-year overall survival. Principal component analysis (PCA) was used to examine the distribution of patients in the two risk groups. Univariate and multivariate Cox regression analyses were conducted to determine whether the anoikis-related risk score was independently associated with overall survival after adjustment for age, sex, grade, and stage.

### Nomogram construction and pathway enrichment analysis

A prognostic nomogram integrating the anoikis-related risk score and clinicopathological variables was constructed using the “rms” R package to predict 1-, 3-, and 5-year OS probabilities. Calibration curves were used to assess the agreement between predicted and observed survival. Gene set enrichment analysis (GSEA) was performed to explore biological pathways differentially enriched between the high- and low-risk groups. The KEGG gene set collection was used as the reference, and pathways with P < 0.01 and false discovery rate q-value < 0.25 were considered significantly enriched.

### Immune infiltration and tumor microenvironment analysis

To evaluate differences in the tumor immune microenvironment between the two risk groups, the CIBERSORT algorithm was used to estimate the relative abundance of 22 tumor-infiltrating immune cell types in LSCC samples. Correlations among immune cell populations were calculated by Pearson correlation analysis. The ESTIMATE algorithm was used to calculate stromal scores, immune scores, and ESTIMATE scores. In addition, single-sample gene set enrichment analysis (ssGSEA) was performed with the “GSVA” R package to compare immune-related functional pathways between high- and low-risk groups. The expression levels of representative immune checkpoint genes were compared between the two subgroups.

### Drug sensitivity analysis

Drug sensitivity was predicted using the oncoPredict R package (version 1.3.1) with the GDSC2 reference matrix as the pharmacogenomic reference. For each TCGA-LSCC sample, the package was used to estimate the half-maximal inhibitory concentration (IC50) of candidate agents from bulk transcriptomic data according to its standard pipeline. Predicted IC50 values were compared between the high- and low-risk groups using the Wilcoxon rank-sum test, with lower predicted IC50 values interpreted as greater predicted sensitivity. These in silico estimates were considered exploratory and were not interpreted as clinical treatment recommendations.

### Single-cell RNA-sequencing analysis

The raw 10x Genomics matrices from GSE290927 were processed using the “Seurat” R package. Cells were retained when they met the following quality-control criteria: nFeature_RNA > 500 and < 7000, nCount_RNA > 500 and < 60000, mitochondrial gene percentage < 20%, and ribosomal gene percentage < 40%. After filtering, 67,596 control cells and 80,736 tumor cells were retained. Gene expression was normalized using LogNormalize with a scale factor of 10,000, and the top 2,000 highly variable genes were selected using the vst method. Data were scaled, principal component analysis was performed, and the first 15 principal components were used for neighbor construction, clustering, and UMAP visualization. Batch effects were inspected and corrected using Harmony when required.

Cell clusters were annotated according to canonical marker genes and manual inspection, including epithelial cells (KRT15, KRT18, KRT19, and EPCAM), T cells (CD2, CD3D, CD3E, and CD3G), B cells (CD19, CD79A, and CD79B), myeloid cells (CD68, LYZ, and LAMP3), NK cells (NCAM1, FCGR3A, KLRD1, TNF, and IFNG), endothelial cells (FLT1, PECAM1, CDH5, and KDR), and fibroblasts (ACTA2, FAP, VIM, FN1, COL1A1, and S100A4). The expression patterns of ANXA5, LCK, PPARG, and SERPINA1 across cell types were visualized by UMAP and violin plots. Anoikis module scores were calculated at the single-cell level using the ARG set and compared between normal and tumor samples.

### Selection of ANXA5 for experimental evaluation

ANXA5 was prioritized from the four-gene signature because (i) it had the largest positive coefficient among the three risk-associated genes, (ii) it was dysregulated between LSCC and adjacent non-cancerous tissues, (iii) its expression was associated with overall survival, and (iv) its biological functions were relevant to cell survival and anoikis-related processes. This prioritization was used to nominate a candidate for focused functional evaluation and did not establish ANXA5 as the only or predominant driver of the signature.

### Cell culture and siRNA transfection

The human LSCC cell line TU686 was provided by the Department of Otolaryngology-Head and Neck Surgery, Huazhong University of Science and Technology. Cells were cultured in Roswell Park Memorial Institute 1640 medium (RPMI 1640) supplemented with 10% fetal bovine serum (FBS; Gibco, Grand Island, NY, USA) and 1% penicillin-streptomycin at 37 °C in a humidified incubator with 5% CO2. For ANXA5 knockdown, cells in the logarithmic growth phase were seeded in 6-well plates and transfected with ANXA5-specific small interfering RNAs (siRNAs) or negative control siRNA using Lipofectamine 3000 (Invitrogen, USA) according to the manufacturer’s instructions. After 6 h, the transfection mixture was replaced with fresh complete medium. Cells were harvested at the indicated time points for quantitative PCR, western blotting, cell viability, apoptosis, and migration assays.

### Immunofluorescence staining

ANXA5 protein expression in LSCC and adjacent normal tissue sections was examined by immunofluorescence staining. Six matched pairs of primary LSCC and adjacent non-tumor tissues were obtained from six patients who underwent surgical resection at Shandong Cancer Hospital and Institute between March 2025 and December 2025. Adjacent non-tumor tissue was sampled at least 0.5cm from the macroscopic tumor margin and was confirmed by histopathological examination to be free of malignant cells. All the patients did not receive preoperative chemotherapy or radiotherapy. The main clinicopathological characteristics, including age, sex, tumor subsite, histological grade, and TNM stage, are summarized in **Supplementary Table 1**. Briefly, tissue sections were deparaffinized, rehydrated, subjected to antigen retrieval, and blocked with serum blocking buffer. Sections were incubated with primary antibody against ANXA5 at 4 °C overnight and then with fluorescence-conjugated secondary antibody at room temperature. Nuclei were counterstained with DAPI. Images were captured using a fluorescence microscope under the same exposure settings for comparison between groups.

### Quantitative real-time PCR and western blotting

Total RNA was extracted from TU686 cells and reverse-transcribed into cDNA using PrimeScript RT reagent Kit (Perfect Real Time; TaKaRa, Beijing, China) according to the manufacturer’s protocol. Quantitative real-time PCR was performed to detect the expression of ANXA5 and epithelial-mesenchymal transition (EMT)-related genes. The primer sequences were as follows: ANXA5 forward 5’-CATCTTTGGGACACGCAG-3’ and reverse 5’-GGTCAATCTCACTCCTC-3’; E-cadherin forward 5’-CGAGAGCTACACGTTCACGG-3’ and reverse 5’-GGGTGTCGAGGGAAAAATAGG-3’; N-cadherin forward 5’-TTTGATGGAGGTCTCCTAACACC-3’ and reverse 5’-ACGTTTAACACGTTGGAAATGTG-3’; Vimentin forward 5’-GACGCCATCAACACCGAGTT-3’ and reverse 5’-CTTTGTCGTTGGTTAGCTGGT-3’. Relative gene expression was calculated using the 2^-ΔΔCt method.

For western blotting, cells were lysed with RIPA buffer containing protease and phosphatase inhibitors (Beyotime, Shanghai, China), and protein concentrations were measured using a BCA Protein Assay Kit (Absin, Shanghai, China). Equal amounts of protein were separated by SDS-PAGE and transferred onto membranes. After blocking, membranes were incubated with primary antibodies against ANXA5 and Vinculin, followed by incubation with appropriate secondary antibodies. Protein bands were visualized using enhanced chemiluminescence (ECL), and band intensities were quantified using ImageJ software (NIH, Bethesda, MD, USA).

### Cell viability assay

Cell viability was evaluated using a Cell Counting Kit-8 (CCK-8; Dojindo, Kumamoto, Japan). Briefly, 5 × 10^3 TU686 cells were seeded into each well of 96-well plates with 100 μL culture medium. At 0, 24, 48, 72, and 96 h after seeding or transfection, 10 μL CCK-8 reagent was added to each well and incubated for 2 h at 37 °C. Absorbance was measured at 450 nm using a microplate reader.

### Cell migration assay

Cell migration was evaluated using a wound-healing assay. TU686 cells were seeded into 6-well plates at a density of 2 × 10^5 cells per well. When cells reached approximately 90% confluence, a straight scratch was created using a sterile 10 μL pipette tip. After gently washing with PBS twice to remove detached cells, serum-free medium was added. Wound areas were photographed at 0 and 24 h under an inverted microscope, and the relative migration percentage was calculated.

### Flow cytometry analysis of apoptosis

Cell apoptosis was detected by flow cytometry. After transfection, TU686 cells were harvested, centrifuged, washed with PBS, and stained using an Annexin V-FITC/7-AAD apoptosis detection kit according to the manufacturer’s instructions. The stained cells were analyzed using a FACS Canto II flow cytometer and Diva software (Becton Dickinson, San Jose, CA, USA). The percentage of apoptotic cells was calculated as the sum of early and late apoptotic populations.

### Statistical analysis

All bioinformatics analyses were performed using R software. Continuous variables between two groups were compared using the Wilcoxon rank-sum test or Student’s t-test, as appropriate. Correlations were assessed using Pearson or Spearman correlation analysis. Kaplan-Meier survival analysis with the log-rank test was used to compare OS between groups. Univariate and multivariate Cox regression analyses were used to evaluate prognostic factors. *In vitro* experiments were performed with at least three independent replicates, and data were presented as mean ± standard deviation. P < 0.05 was considered statistically significant.

## Results

### Identification of anoikis-related differentially expressed genes in LSCC

In the analysis of anoikis-related differentially expressed genes (ARDEGs) in LSCC, we first calculated the anoikis-related score in the GSE290927 single-cell RNA-seq dataset using the single-cell anoikis module score function based on the 640 candidate ARGs obtained from the GeneCards and Harmonizome databases. The anoikis-related score was significantly lower in LSCC tissues than in non-tumor tissues (**Figure 1A**), suggesting attenuated anoikis-related activity and potential enhancement of anoikis resistance in malignant tissues. Subsequently, differential expression analysis was performed based on the TCGA-LSCC cohort to identify ARGs that were significantly dysregulated between LSCC and adjacent normal tissues, using |log2FC| > 1 and adjusted P < 0.05 as screening criteria. These differentially expressed ARGs were visualized using volcano plot and heatmap analyses (**Figures 1B, C**). Through univariate Cox regression analysis, 16 anoikis-related DEGs were associated with overall survival, including one protective factor (LCK) and 15 risk factors (EGFR, ITGA5, PIK3CA, ANXA5, PLAUR, PDGFB, SERPINA1, INHBB, PAK2, SIRPA, MYH9, BAG4, SERPINE1, and FOXC2) (**Figure 1D**). Subsequently, least absolute shrinkage and selection operator (LASSO) Cox regression was applied to refine the candidates and reduce overfitting. The optimal lambda (lambda) value was determined by 10-fold cross-validation, and the corresponding coefficient profile supported further screening of prognostic genes (**Figures 1E, F**). Finally, a coexpression network was constructed among the screened candidate genes, illustrating their coordinated expression relationships in LSCC (**Figure 1G**).

**Figure 1:**
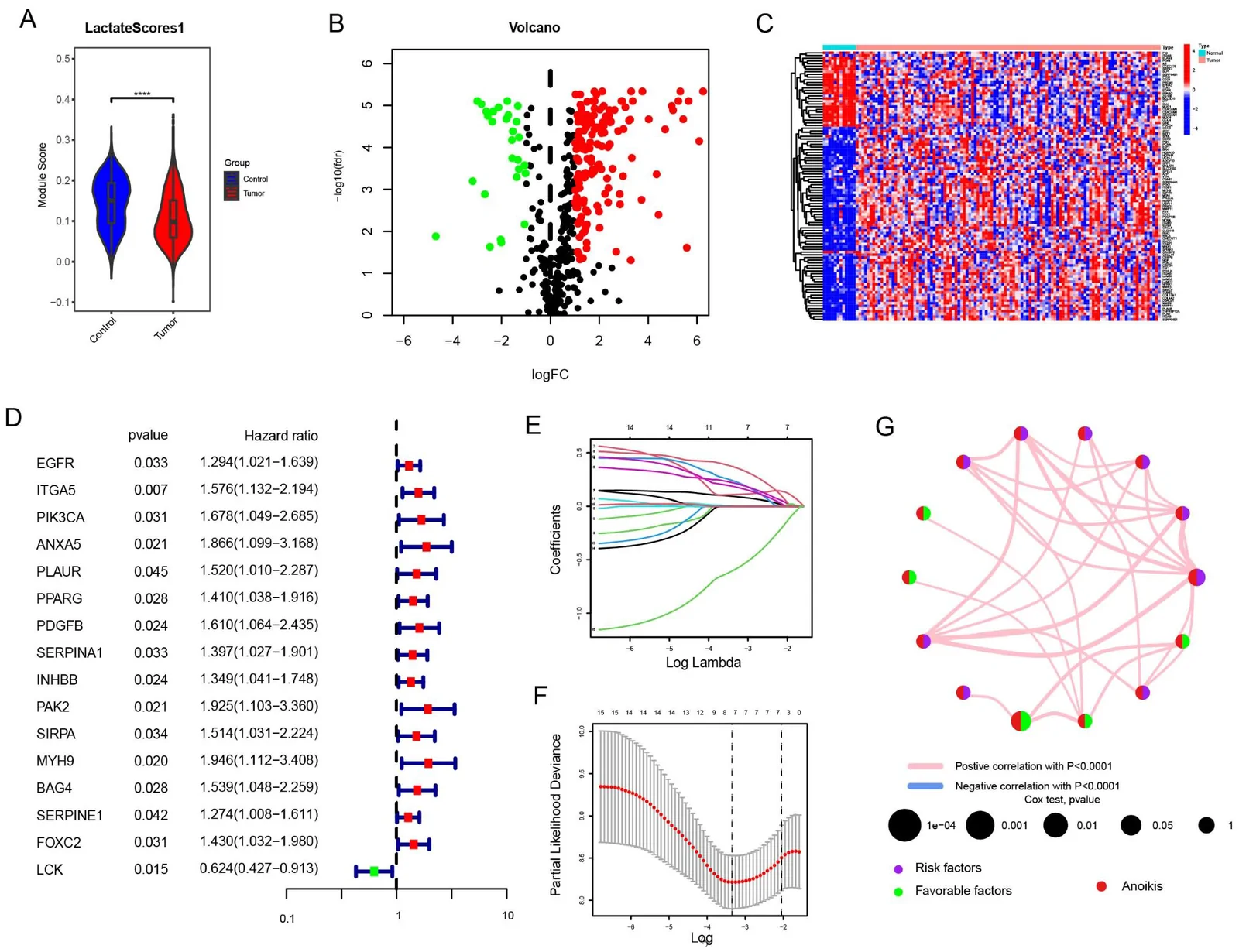
Identification of anoikis-related differentially expressed genes in laryngeal squamous cell carcinoma (LSCC). **(A)** Anoikis-related score analysis in the GSE290927 single-cell RNA-seq dataset using the single-cell anoikis module score function based on the 640 candidate anoikis-related genes. The anoikis-related score was compared between cells from LSCC tissues and non-tumor tissues. **(B)** Volcano plot of differentially expressed anoikis-related genes from the TCGA-LSCC cohort. **(C)** Heatmap of upregulated and downregulated genes between LSCC and normal tissues. **(D)** Univariate Cox regression analysis screening prognostic anoikis-related differentially expressed genes. **(E, F)** LASSO Cox regression coefficient profile and cross-validation curve. **(G)** Coexpression network of candidate anoikis-related genes.

### Construction and evaluation of the anoikis-related gene signature

To evaluate the performance and robustness of the anoikis-related prognostic signature, patients were stratified into high-risk and low-risk groups based on the median risk score in the training set, and the same cutoff was subsequently applied to the testing set and the entire TCGA-LSCC cohort. Heatmap analysis showed distinct expression patterns of the four signature genes (ANXA5, PPARG, SERPINA1, and LCK) between the high-risk and low-risk subgroups across all three datasets (**Figure 2A**). Risk-score distribution plots showed a clear gradient when patients were ordered by increasing risk score, and patients in the high-risk group had higher mortality than those in the low-risk group (**Figure 2B**). Further analysis confirmed a positive association between the calculated risk score and mortality probability (**Figure 2C**). Kaplan-Meier survival analysis demonstrated significant differences in overall survival between the high-risk and low-risk groups in the training, testing, and entire TCGA cohorts (**Figure 2D**), with poorer survival in high-risk patients. ROC curves showed that the risk score had favorable discriminatory ability compared with conventional clinical variables, with AUC values of 0.722, 0.747, and 0.706 in the training, testing, and entire cohorts, respectively (**Figure 2E**). Time-dependent ROC analyses further supported the predictive performance of the signature for 1-, 3-, and 5-year overall survival (training: 0.747, 0.849, and 0.834; testing: 0.706, 0.810, and 0.722; entire cohort: 0.722, 0.845, and 0.835) (**Figure 2F**).

**Figure 2:**
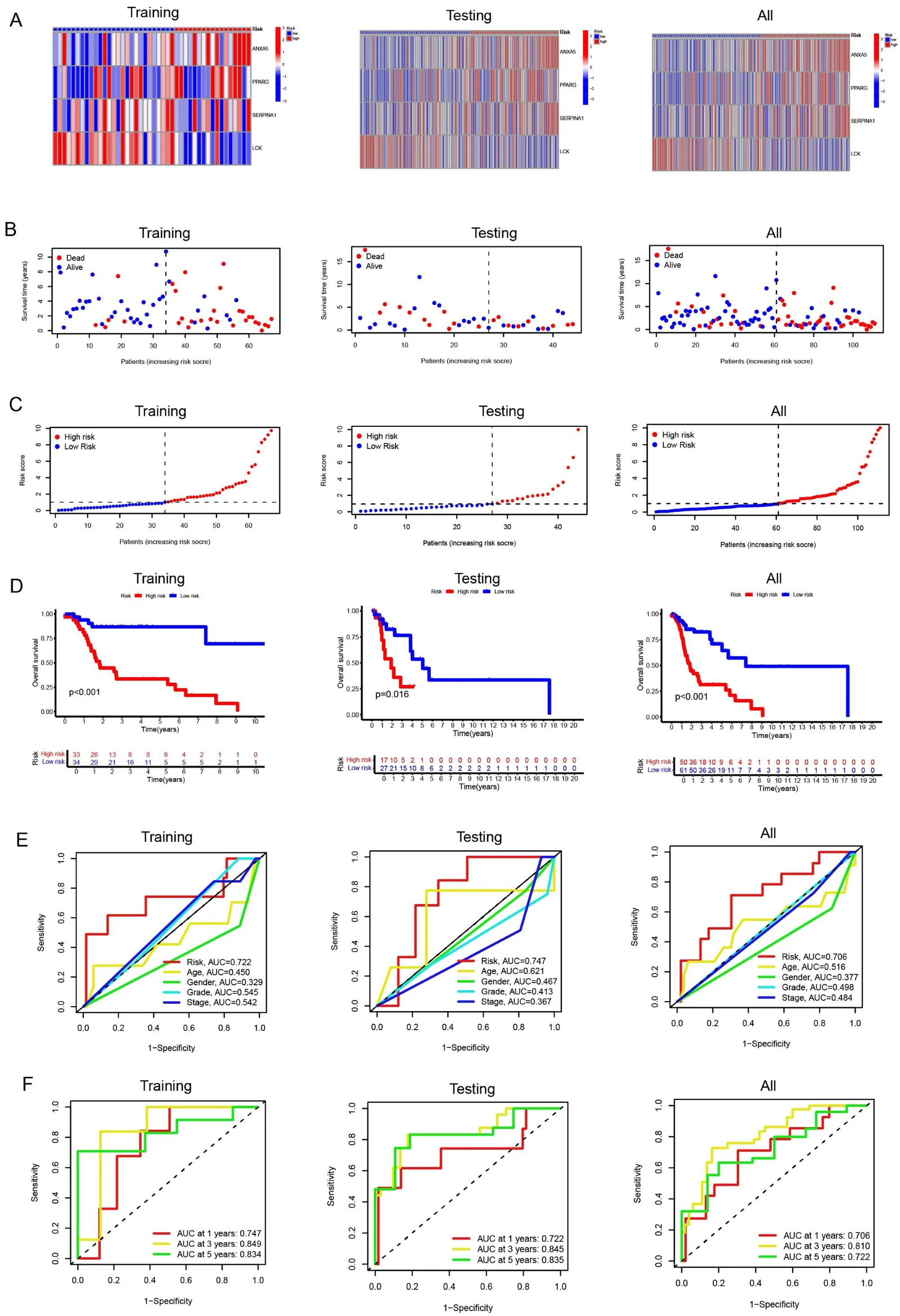
Construction and evaluation of anoikis-related gene signature model in the training, testing and all sets. **(A)** Heatmap illustrating the expression profiles of four signature genes in high-risk versus low-risk subgroups across the training, testing, and entire TCGA cohorts. Red and blue indicate relatively high and low expression levels, respectively. **(B)** Risk score distribution and corresponding patient survival status. Patients are ordered by ascending risk score, revealing a clear trend: individuals in the high-risk group (red) display markedly higher mortality compared to those in the low-risk group (blue). **(C)** Scatter plot demonstrating the positive correlation between risk score and mortality probability in LSCC patients. **(D)** Kaplan-Meier curves illustrating overall survival differences between the high-risk and low-risk subgroups in every cohort. **(E)** Receiver operating characteristic (ROC) curves assessing the discriminatory ability of the risk score model in comparison with conventional clinical parameters. **(F)** Time-dependent ROC analyses evaluating the model’s predictive performance for overall survival at 1-year, 3-year, and 5-year landmarks.

### Independent prognostic value of the anoikis-related risk score in LSCC

To further assess the independent prognostic value of the anoikis-related gene signature, univariate and multivariate Cox proportional hazards regression analyses were performed using the entire TCGA-LSCC cohort. In univariate analysis, the risk score was significantly associated with overall survival (HR = 1.403, 95% CI: 1.226-1.605, P < 0.001) (**Figure 3A**). After adjustment for age, gender, grade, and stage, the risk score remained an independent prognostic factor (HR = 1.483, 95% CI: 1.242-1.771, P < 0.001), while stage also retained prognostic significance in the multivariate model (HR = 2.863, 95% CI: 1.128-7.265, P = 0.027) (**Figure 3A**). Principal component analysis showed separation between the high-risk and low-risk groups in the training, testing, and entire TCGA cohorts (**Figure 3B**). A nomogram integrating the risk score with age, gender, grade, and stage was constructed to predict 1-, 3-, and 5-year overall survival probabilities in LSCC patients (**Figure 3C**). Calibration curves demonstrated good agreement between predicted and observed survival, with a C-index of 0.771 (95% CI: 0.704-0.838) (**Figure 3D**). Gene set enrichment analysis revealed that the high-risk group was enriched in KEGG arrhythmogenic right ventricular cardiomyopathy, dilated cardiomyopathy, ECM-receptor interaction, focal adhesion, and hypertrophic cardiomyopathy pathways. In contrast, the low-risk group was enriched in allograft rejection, asthma, autoimmune thyroid disease, primary immunodeficiency, and type I diabetes mellitus pathways (**Figure 3E**).

**Figure 3:**
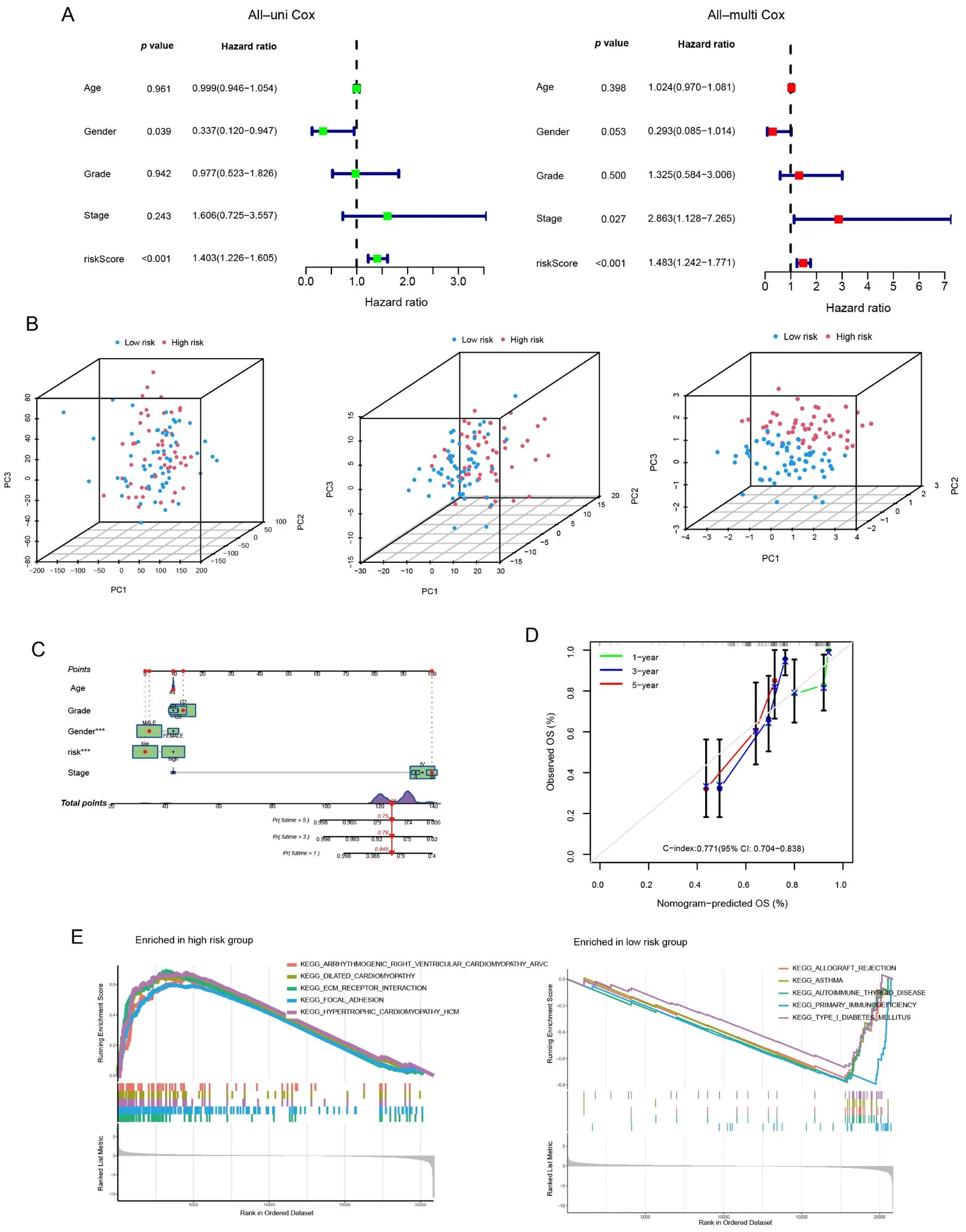
Independent prognostic value and functional enrichment of the anoikis-related risk score in laryngeal squamous cell carcinoma. **(A)** Univariate and multivariate Cox regression analyses in the TCGA cohort. **(B)** Principal component analysis demonstrating separation between high-risk and low-risk groups in the training, testing, and entire TCGA cohorts. **(C)** Nomogram integrating the anoikis-related risk score with clinical factors to predict 1-, 3-, and 5-year overall survival. **(D)** Calibration curve evaluating the predictive accuracy of the nomogram. **(E)** Gene set enrichment analysis showing KEGG pathways enriched in the high-risk and low-risk groups.

### Immune microenvironment analysis and drug sensitivity evaluation

To investigate the association between the anoikis-related gene signature and the tumor immune microenvironment in laryngeal squamous cell carcinoma (LSCC), we compared immune cell infiltration patterns between the high-risk and low-risk groups across the TCGA cohort.

Bar plot analysis revealed distinct immune cell infiltration profiles between the two risk groups (**Figure 4A**). The correlation heatmap illustrated interrelationships among infiltrating immune cell types in LSCC (**Figure 4B**). Violin plots further confirmed statistically significant differences in key immune subsets: the low-risk group displayed higher proportions of plasma cells, CD8+ T cells, activated memory CD4+ T cells, follicular helper T cells, and regulatory T cells, whereas the high-risk group was characterized by increased resting memory CD4+ T cells, M0 macrophages, and M2 macrophages (**Figure 4C**). ESTIMATE analysis showed that the low-risk group had a significantly higher immune score, indicating stronger immune infiltration than the high-risk group (**Figure 4D**).

**Figure 4:**
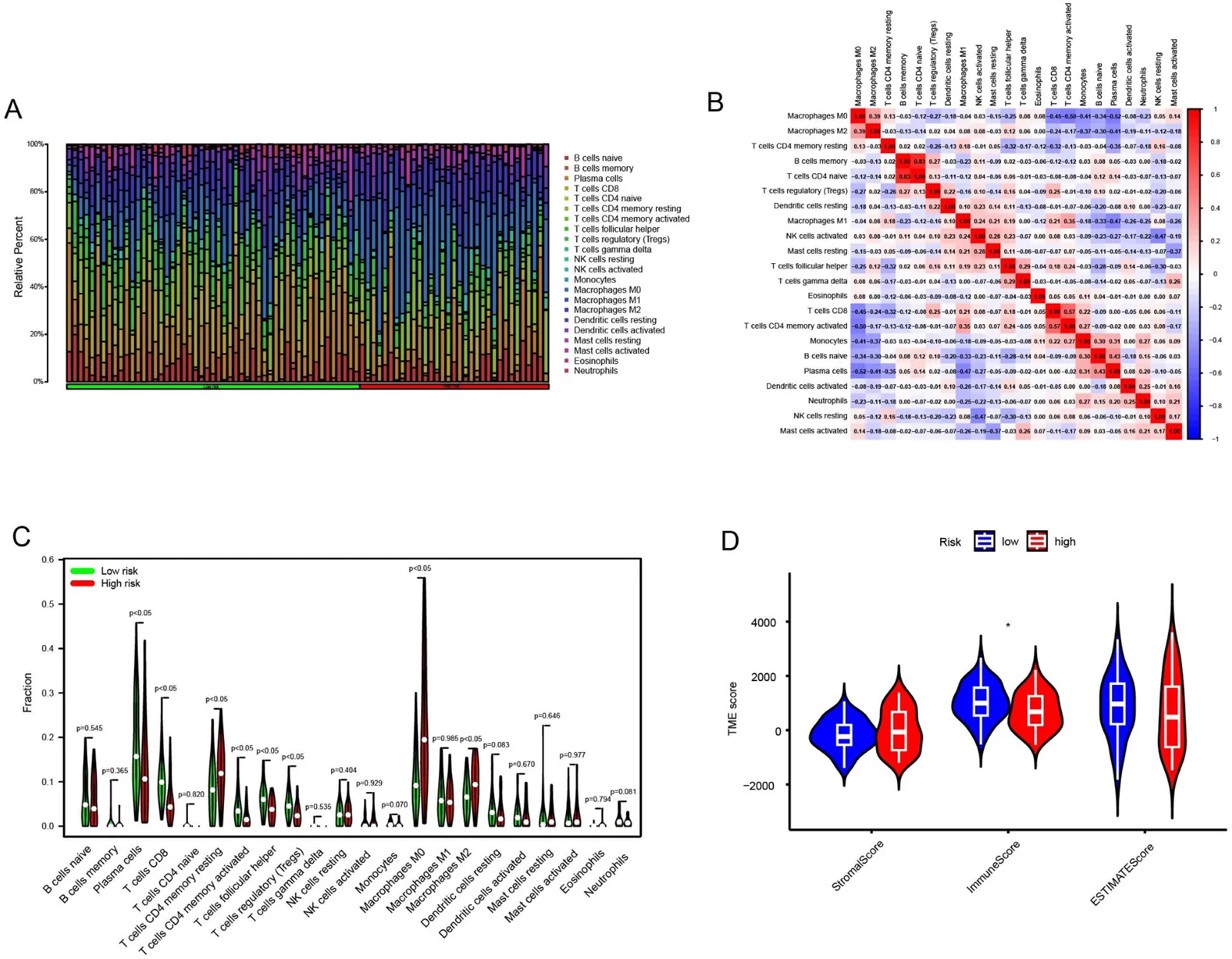
Immune cell infiltration analysis in laryngeal squamous cell carcinoma patients. **(A)** Distribution of immune cell infiltration between high-risk and low-risk groups based on the anoikis-related gene signature. **(B)** Correlations among immune cells in LSCC patients. **(C)** Violin plots showing immune cell composition in the high-risk and low-risk groups. **(D)** Stromal, immune, and ESTIMATE scores in the high-risk and low-risk groups. *P < 0.05, **P < 0.01, ***P < 0.001. ns, no significance.

Single-sample gene set enrichment analysis revealed marked differences in immune functional pathways between the risk groups. The low-risk group showed higher activity in several anti-tumor immune processes, including APC co-stimulation, cytolytic activity, HLA, inflammation-promoting response, T-cell co-stimulation, and type I IFN response (**Figure 5A**). Expression levels of immune checkpoint-related molecules also differed between the two groups; several genes, including CD44, PDCD1LG2, CD276, and NRP1, were increased in the high-risk group, whereas other immune regulators showed opposite trends (**Figure 5B**). Drug sensitivity prediction indicated that the high-risk group displayed significantly higher predicted response to cyclophosphamide, paclitaxel, sorafenib, and zoledronate (**Figure 5C**).

**Figure 5:**
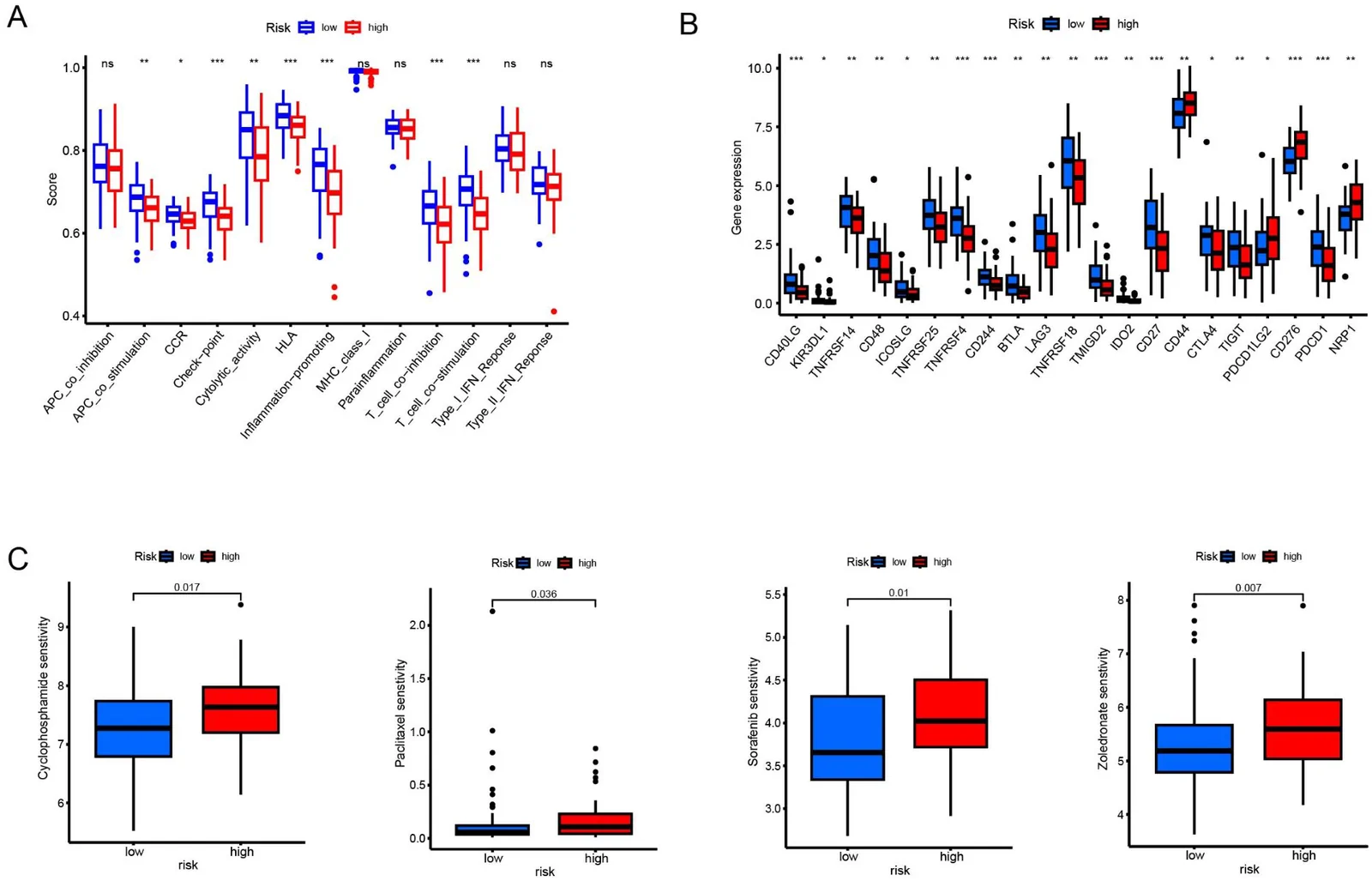
Immune function, checkpoint expression, and drug sensitivity analyses in high-risk and low-risk groups. **(A)** Immune functional differences between the high-risk and low-risk groups based on ssGSEA scores. **(B)** Expression levels of immune checkpoint-related genes in the two groups. **(C)** Chemotherapy drug sensitivity analysis between the two groups. *P < 0.05, **P < 0.01, ***P < 0.001.

### Single-cell analysis of anoikis-related signature genes in LSCC

To further elucidate the cellular origin and heterogeneity of the anoikis-related prognostic signature, single-cell RNA sequencing data from the GSE290927 dataset were analyzed using UMAP dimensionality reduction and clustering. UMAP visualization identified seven major cell populations, including T cells, endothelial cells, fibroblasts, NK cells, epithelial cells (EpCs), myeloid cells, and B cells (**Figure 6A**). The distribution of the four core genes (ANXA5, LCK, PPARG, and SERPINA1) across these cell populations was then examined (**Figure 6B**). ANXA5 was prominently expressed in fibroblasts, NK cells, and myeloid cells, with additional expression in epithelial cells. LCK was mainly expressed in T cells, PPARG was enriched in NK cells, and SERPINA1 was mainly expressed in NK and myeloid cells (**Figure 6B**). Bulk expression analysis showed that ANXA5, LCK, PPARG, and SERPINA1 were differentially expressed between normal and tumor tissues (**Figure 6C**). Kaplan-Meier survival analysis of individual signature genes further showed that expression-based stratification of ANXA5, LCK, PPARG, and SERPINA1 was associated with overall survival in LSCC patients (**Figure 6D**).

**Figure 6:**
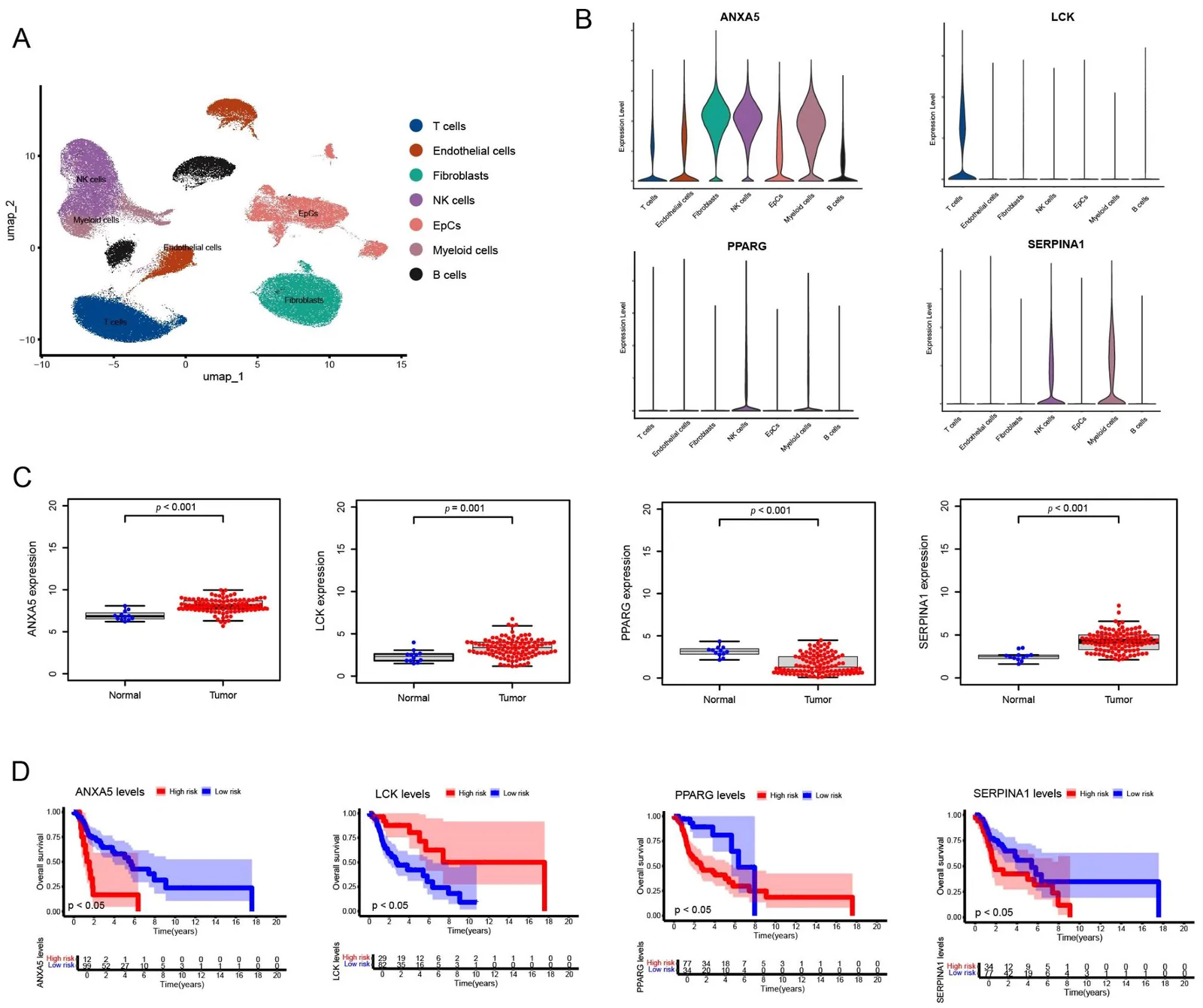
Single-cell analysis of anoikis-related genes in laryngeal squamous cell carcinoma (LSCC, GSE290927). **(A)** UMAP clustering of all cells from GSE290927 showing seven major cell populations. **(B)** Distribution of the four key genes across different cell clusters in single-cell analysis. **(C)** Gene expression levels of the four key genes (ANXA5, LCK, PPARG, SERPINA1) in normal tissues compared with LSCC patient samples. **(D)** Kaplan-Meier survival analysis showing the relationship between individual key-gene expression and overall survival in LSCC patients.

### *In vitro* functional characterization of ANXA5 in LSCC

ANXA5 was prioritized for experimental evaluation from the four-gene signature because it had the largest positive coefficient among the three risk-associated genes, was dysregulated between LSCC and adjacent non-cancerous tissues, was associated with overall survival, and had biological relevance to cell-survival and anoikis-related processes. This prioritization nominated ANXA5 as a candidate for focused functional study but did not establish it as the only or predominant driver of the signature.

To investigate the biological role of ANXA5 in LSCC, we first examined its protein expression in tumor tissues and matched adjacent normal tissues from six LSCC patients using immunofluorescence staining (**Figure 7A**). ANXA5 exhibited stronger staining in LSCC tumor tissues than in adjacent normal tissues, confirming its upregulation at the protein level in malignant tissues. ANXA5 knockdown was then performed in TU686 cells using specific siRNAs. Successful silencing was verified by qRT-PCR and Western blot analysis, which demonstrated reduced ANXA5 mRNA and protein levels compared with negative control siRNA-transfected cells (**Figures 7B, C**). Functional assays revealed that ANXA5 knockdown impaired malignant phenotypes. CCK-8 assay showed that cell viability was significantly decreased in ANXA5-knockdown cells, especially at later time points (**Figure 7D**). Knockdown of ANXA5 increased E-cadherin expression and decreased N-cadherin and Vimentin expression (**Figure 7E**), suggesting that ANXA5 may participate in sustaining mesenchymal features in LSCC cells. Flow cytometry analysis further revealed that ANXA5 knockdown increased the proportion of apoptotic cells compared with the control group (**Figure 7F**), indicating that ANXA5 supports cell survival. Finally, wound-healing assays demonstrated that ANXA5 silencing markedly suppressed wound closure at 24 h (**Figure 7G**), hinting that ANXA5 might facilitate the migratory capability.

**Figure 7:**
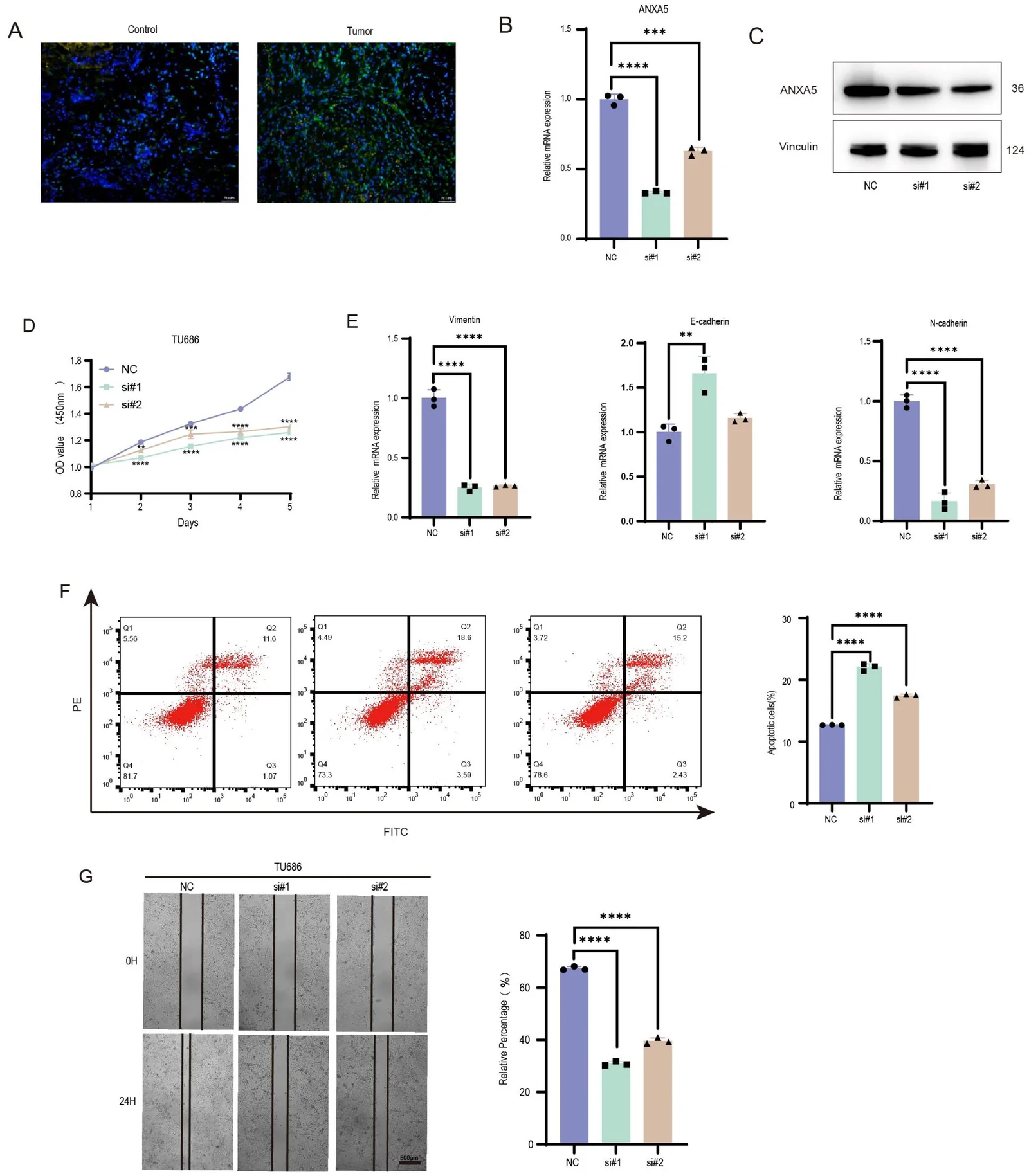
*In vitro* study of the effects of ANXA5 on laryngeal squamous cell carcinoma. **(A)** Representative images of ANXA5 immunofluorescence staining in six matched LSCC/adjacent non-tumor pairs from the same patients **(B)** Detection of ANXA5 mRNA expression in TU686 cells after ANXA5 knockdown. **(C)** Western blot evaluation of ANXA5 protein expression after ANXA5 knockdown. **(D)** Assessment of TU686 cell viability after ANXA5 knockdown. **(E)** Gene expression levels of epithelial-mesenchymal transition-related markers (E-cadherin, N-cadherin, and Vimentin) after ANXA5 knockdown. **(F)** Flow cytometry analysis of apoptosis rates after ANXA5 knockdown. **(G)** Wound-healing assay performed 24 h after ANXA5 knockdown in TU686 cells.

## Discussion

Anoikis, a specialized form of apoptosis triggered by detachment from the extracellular matrix, serves as a critical barrier against cancer metastasis (26, 27). Resistance to anoikis is a hallmark of malignant progression in various cancers, including head and neck squamous cell carcinoma, where it facilitates tumor cell survival in circulation and distant seeding (28–30). In LSCC, a major subtype of HNSCC, impaired anoikis mechanisms contribute significantly to aggressive behavior, poor prognosis, and therapeutic resistance (31). Our study systematically identified ARDEGs in LSCC using GSE290927 for anoikis scoring and TCGA-LSCC for differential expression and prognostic modeling. The significantly lower anoikis enrichment score in tumor tissues aligns with prior observations of anoikis dysregulation in HNSCC, underscoring its role in promoting detachment survival and metastasis.

Through univariate Cox regression, we identified 16 prognostic ARDEGs, with LCK as the sole protective factor and 15 risk factors (including EGFR, ITGA5, PIK3CA, ANXA5, among others). LASSO regression refined this to a robust four-gene signature (ANXA5, LCK, PPARG, SERPINA1), which demonstrated strong prognostic performance across training, testing, and entire TCGA cohorts. Patients stratified into high-risk groups consistently exhibited poorer overall survival, independent of conventional clinicopathological factors, as confirmed by multivariate Cox analysis and PCA-based separation. The constructed nomogram integrating the risk score with age, gender, grade, and stage showed excellent calibration, highlighting its potential for individualized risk assessment in clinical practice.

GSEA identified several pathways with cardiomyopathy-related KEGG labels in the high-risk group. In the context of LSCC, these enrichments likely reflect overlap in extracellular-matrix, focal-adhesion, cytoskeletal, and contractile gene components shared across biological systems rather than cardiac disease processes. Accordingly, the pathway names should be interpreted as annotations of overlapping gene programs and not as evidence of cardiomyopathy or specific metabolic remodeling in LSCC. The immune-related pathways enriched in the low-risk group, together with the immune-infiltration results, suggest differences in immune-associated transcriptional programs; however, causal relationships cannot be inferred from enrichment analysis alone.

Immune microenvironment analysis further supported this notion. The low-risk group exhibited higher infiltration of anti-tumor effectors (CD8+ T cells, activated CD4+ memory T cells, follicular helper T cells) and elevated immune/ESTIMATE scores, alongside enhanced cytolytic activity, IFN responses, and co-stimulation pathways via ssGSEA. In contrast, the high-risk group showed immunosuppressive features, including increased M2 macrophages, resting CD4+ memory T cells, and upregulated checkpoint molecules (TNFRSF18, CD44, PDCD1LG2, CD276, NRP1). This immunosuppressive landscape in high-risk patients may explain relative chemoresistance to certain agents, while paradoxically showing sensitivity to cyclophosphamide, paclitaxel, and sorafenib—findings that warrant valuation in functional drug screens and could guide personalized therapy selection.

Single-cell analysis demonstrated substantial cell-type heterogeneity of the signature genes. ANXA5 was prominently expressed in fibroblasts, NK cells, and myeloid cells, with additional expression in epithelial cells; LCK was mainly expressed in T cells, PPARG in NK cells, and SERPINA1 in NK and myeloid cells. Therefore, ANXA5 may have context-dependent functions in malignant epithelial, stromal, and immune populations. The TU686 experiments specifically support a functional role in an LSCC epithelial tumor-cell model but do not demonstrate that epithelial cells are the exclusive or predominant tissue source of ANXA5. Cell-type-specific perturbation studies will be required to define its roles in fibroblasts and immune populations and their contribution to stromal–immune–tumor crosstalk.

Functional characterization focused on ANXA5, the most prominent risk factor, confirmed its overexpression in LSCC tissues via immunofluorescence. Knockdown in TU686 cells reduced viability, enhanced apoptosis (supporting its anti-anoikis role), changed mesenchymal molecular markers (upregulated E-cadherin, downregulated N-cadherin/Vimentin), and inhibited migration, demonstrating that ANXA5 actively promotes proliferation, mesenchymal traits, anoikis resistance, and invasiveness in LSCC. These *in vitro* results corroborate bioinformatics predictions and position ANXA5 as a potential candidate biomarker, consistent with recent reports linking ANXA5 to proliferation, migration, immune modulation, and poor prognosis in HNSCC and oral squamous cell carcinoma.

This study has several limitations. First, model development and testing used random subsets of the same relatively small TCGA-LSCC cohort (111 tumors), so the testing set represents internal rather than external validation. No independent survival cohort was included. Second, tissue-level validation involved only six matched pairs and should be considered preliminary. Third, functional experiments were performed in a single LSCC cell line, and apoptosis was assessed under standard adherent conditions rather than in a dedicated detachment-induced anoikis model; therefore, validation in additional cell lines, anchorage-independent assays, *in vivo* models, and patient-derived systems is required. Fourth, single-cell data showed ANXA5 expression in several stromal and immune populations, whereas the present experiments examined only tumor epithelial cells. Fifth, drug-response results were generated computationally from public pharmacogenomic reference data and were not validated experimentally or clinically. Potential batch effects and incomplete clinical annotation in public datasets may also influence the results. Finally, *in-vitro* functional assays support the pro-malignant roles of ANXA5 in regulating cell proliferation, apoptosis, epithelial-mesenchymal transition and migratory capacity, the present study is limited to cell-based observations. Further investigations are needed to identify key downstream signaling cascades that account for these phenotypic alterations. In addition, *in-vivo* animal models should be implemented to validate the biological functions of ANXA5 under physiological tumor-bearing conditions. In Future work, we will further dissect the underlying molecular mechanisms mediating the oncogenic effects of ANXA5 in LSCC. In addition, in-vivo animal models should be implemented to validate the biological functions of ANXA5.

## Conclusion

Within the TCGA-LSCC cohort, the four-gene anoikis-related signature stratified overall survival and was associated with differences in the tumor immune microenvironment. ANXA5 knockdown altered viability, apoptosis, EMT-marker expression, and migration in TU686 cells, supporting its relevance to malignant epithelial-cell phenotypes. These findings nominate the signature and ANXA5 for external prognostic validation and additional cell-type-specific and mechanistic studies; they should not yet be used to guide treatment selection.

## Data Availability

The raw data supporting the conclusions of this article will be made available by the authors, without undue reservation.
